# The Botany, &c. of the Himalayan Mountains

**Published:** 1837-01

**Authors:** 


ART. VIII.—*Illustrations of the Botany, &c. of the Himalayan Mountains and Cashmere.* By T. FORBES ROYLE, Esq. Parts 7, 8, 9.—Pp. 217-331. Folio. *London*, 1835-6.

THE elaborate details of the press and plates of this work justly commend the author's researches to the attention of the scientific reader; in notices upon medical botany also these numbers are peculiarly rich. They present also another striking feature, exhibiting the infinitely varied supplies of vegetable produce available to the wants of man over all the earth. These addenda to modern works on natural history evince the advanced state to which such investigations have arrived; and we accept the author's observations (pp. 310, 311,) upon the deficiency experienced in existing views of botanical geography, as an earnest of increasing attention to so important a branch of the science. The eighth part contains an interesting article, too concisely written perhaps, upon the attempts made to cultivate the tobacco plant in our eastern colonies. We regret to find the general failure of these attempts confirmed by the author's report; but we must refer the reader to the article itself. We cannot, however, resist noticing the ingenious expedient of the hooqqualess mountaineer, who is still able to luxuriate in the fumes of the delicious weed by roasting it in a hole in the ground, (p. 282,) we conclude, "*non sine fistula.*" Plates 85 and 86 deserve more careful colouring.

